# Multimodal therapy strategies based on hydrogels for the repair of spinal cord injury

**DOI:** 10.1186/s40779-022-00376-1

**Published:** 2022-04-12

**Authors:** Yan Wang, Hong-Qian Lv, Xuan Chao, Wen-Xin Xu, Yun Liu, Gui-Xia Ling, Peng Zhang

**Affiliations:** 1 Shenyang Pharmaceutical University, 103 Wenhua Road, Shenyang, 110016 China; 2 Center for Nanotechnology in Drug Delivery, Eshelman School of Pharmacy, University of North Carolina at Chapel Hill, Chapel Hill, NC 27599 USA

**Keywords:** Spinal cord injury, Injectable hydrogels, Hydrogel scaffolds, Multimodal therapy

## Abstract

Spinal cord injury (SCI) is a serious traumatic disease of the central nervous system, which can give rise to the loss of motor and sensory function. Due to its complex pathological mechanism, the treatment of this disease still faces a huge challenge. Hydrogels with good biocompatibility and biodegradability can well imitate the extracellular matrix in the microenvironment of spinal cord. Hydrogels have been regarded as promising SCI repair material in recent years and continuous studies have confirmed that hydrogel-based therapy can effectively eliminate inflammation and promote spinal cord repair and regeneration to improve SCI. In this review, hydrogel-based multimodal therapeutic strategies to repair SCI are provided, and a combination of hydrogel scaffolds and other therapeutic modalities are discussed, with particular emphasis on the repair mechanism of SCI.

## Background

Spinal cord injury (SCI) is a debilitating systemic disease of the central nervous system (CNS) and one of the most severe traumatic diseases. It influences thousands of people each year and most of them are young people [1]. It leads to a gradual loss of motor and sensory function, which poses a threat to the patients’ health and seriously affects the life expectancy of patients. Long-term treatment, care costs and financial losses can affect patients and their families, causing social and physical problems [2]. However, effective treatments for SCI have not been developed to date. With the current advance of biomaterials applications in medicine, many studies suggested the potential of biomaterial for SCI repairment. Hydrogels as a biomaterial have a good application in the nervous system due to their properties of hydrophilicity and biocompatibility [3].

Hydrogels are highly hydrated materials with water molecules and hydrophilic polymer networks. They have become one of the most conspicuous biological materials and their intrinsic properties such as biocompatibility, cell interactions, hydrophilic, permeability and biodegradation make them become suitable substrates to imitate the natural molecular microenvironment [4, 5]. Hydrogels are usually made of natural or synthetic materials. Natural hydrogels are divided into protein and polysaccharides, including collagen, gelatin, chitosan, alginate, agarose, etc. Natural polymer hydrogels are good candidates for the treatment of SCI due to the biocompatible and biodegradable nature. Synthetic hydrogels are made from artificial materials such as methacrylate and polyethylene glycol, which limit biological activity and have side-effects, such as cell adhesion [6]. But the biological properties can be improved by mixing with natural biomaterials or natural polymers, and thus these synthetic hydrogels also exhibit the required biocompatibility and biodegradability. A wide variety of natural hydrogels and synthetic hydrogels have different polymer topologies and chemical compositions, which make them highly adaptable for a wide application, such as conductive coating of nerve electrode [5, 7], regenerative biomaterial [8, 9], drug delivery [10], sensing [11], lubrication and so on [12–17]. Hydrogels have been widely utilized as SCI repair materials, and achieved a relatively good effect for SCI improvement in recent years. Moreover, the stem cells-loaded hydrogel therapy has shown that it can enhance the therapeutic efficacy for SCI treatment [18, 19]. When hydrogels are used as scaffolds for transplantation of stem cells, the cytocompatibility of hydrogels is a very important factor, and it is necessary to ensure that hydrogels can promote the survival rate of transplanted and increase therapeutic effect. Hydrogels act like the extracellular environment of body tissues, making them useful for cell transplantation. Hydrogels with cytocompatibility tend to influence their interactions with cell systems by controlling their physical properties and biological activities [20].

**Fig. 1:**
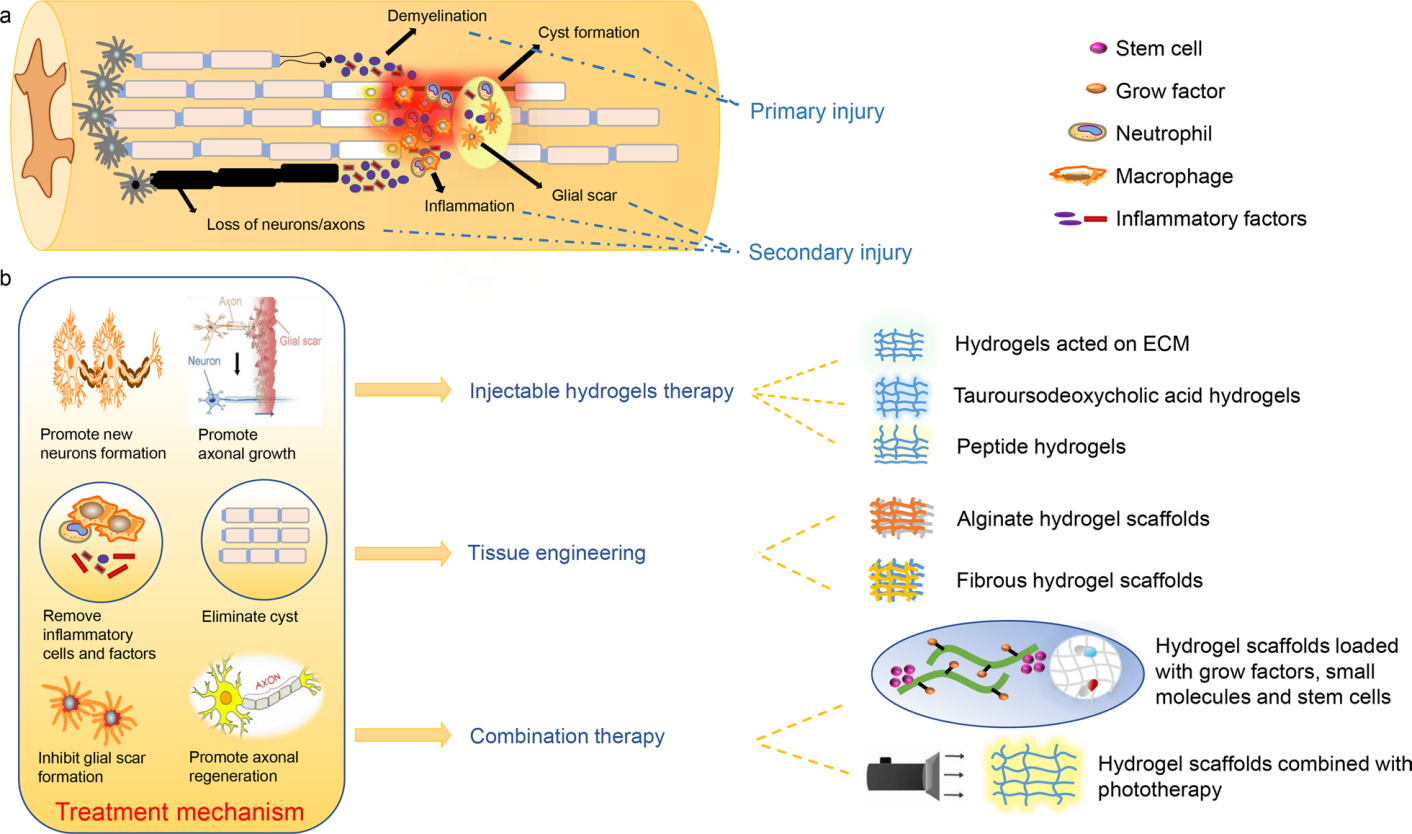
Mechanisms of spinal cord injury (SCI) and different models therapy based on hydrogels. **a** A range of pathologic mechanisms following SCI: primary injury including demyelination and cyst formation. Secondary injury including loss of neurons/axons, inflammation and glial scar. **b** Multimodal therapy strategies based on hydrogel: injectable hydrogels therapy, tissue engineering and combination therapy. The therapy mechanisms based on hydrogels: promote new neurons formation, promote axonal growth, remove inflammatory cells and factors, eliminate cyst, inhibit glial scar formation, promote axonal regeneration. ECM extracellular matrix

At present, hydrogels have been widely applied in the repair of SCI [6, 21]. However, few articles comprehensively overviewed the applications of various hydrogels in repairing SCI. This paper comprehensively reviews hydrogel-based multimodal therapy strategies for the repair of SCI, including injectable hydrogels therapy, tissue engineering (alginate hydrogel scaffolds, and fibrous hydrogel scaffolds) and combination therapy of hydrogel scaffolds combined with phototherapy, growth factors (GFs), small molecules and stem cells (Fig. 1).

## SCI

### Pathological mechanisms of SCI

Spinal cord, as one of the components of the CNS, plays a key role in the regulation of vital activities. Therefore, when SCI occurs, it can give rise to complex pathophysiology which affects the nerves, blood vessels and immune system and a loss of motor and sensory function, loss of autoregulation of breathing, and dysfunction of intestinal regulation, leading to permanent disability. Complex pathological change occurs in the central spinal cord after contusion, compression, or traumatic accident [1]. Spinal cord compression is the most common form of SCI and persists after injury [2]. It can lead to spinal canal hematomas. SCI is associated with bleeding in the early stages, followed by interruptions in the blood supply, which can lead to hypoxia and ischemic infarcts, both of which can damage gray matter with high metabolism and neurons and decrease the thickness of myelin, leading to a host of pathological changes [2].

SCI is characterized by mechanical damage of neurons and glial membranes, microvascular destruction, abnormal ion regulation, and proapoptotic signaling caused by initial traumatic injury, which often cause secondary damage cascade including activation of inflammatory cells, neurons and glial cells apoptosis, collagen fiber acidic protein expression, axonal injury and glial scar formation [22–24]. The secondary injury can extend the damage to areas which are far from the center of the injury and aggravated SCI. Moreover, SCI often has a wide range of inflammatory responses induced by macrophages, neutrophils, T cells and a range of pro-inflammatory factors which can cause lesion growth and tissue damage. Each model has slight differences in the degree of inflammatory infiltration, which hinders tissue regeneration. The functional loss caused by SCI is often lasting because of the limited regenerative ability of damaged neurons and the diffuse lesion site [2, 25].

### Therapy of SCI

Currently, the treatments of SCI in the clinical have some limitations. There are two primary strategies for SCI. The first strategy is to protect the remaining axons and neurons from secondary injury by early clinical decompression or elimination of secondary inflammation in the acute stage of injury. Specific treatment measures include high dose methylprednisolone, calcium channel antagonist, naloxone and other drugs, or local hypothermia protection, artificial high-pressure perfusion, etc. However, the treatment effect is limited with a poor prognosis. The second strategy is cell-based therapy to promote the repair and regeneration of nerve tissue in the chronic period of injury, including surgical treatment, transplantation of stem cells and hyperbaric oxygen therapy. Although good results have been achieved, the survival, migration and attachment of cells in cerebrospinal fluid (CSF) still pose great challenges [22]. Therefore, the key to treating SCI is anti-inflammatory, replacing damaged spinal tissue and promoting regeneration. The anti-inflammatory effect can be achieved by removing inflammatory cells and inhibiting the expression of proinflammatory factors. Replacing damaged spinal tissue can be achieved by inhibiting the formation of glial scar and promoting the repair of damaged neurons and the formation of new neurons. Axonal growth and regeneration can promote spinal cord regeneration.

Although a lot of resources and effort have been put into the search for effective treatments of SCI, there is no active or permanent cure for SCI. Anti-inflammation, replacing damaged spinal tissue and regeneration are the primary targets in the treatment of SCI. Based on the context, novel therapy strategies including injectable hydrogels therapy, tissue engineering and a combination therapy based on hydrogel scaffolds have been applied to the SCI repair. The most common therapy is tissue engineering which focuses on the delivery of specific growth neurotrophic factors [26], stem cells and anti-inflammatory agents to the specific injury site of the SCI delivered by a biomaterial scaffold.

## Injectable hydrogels therapy

In general, hydrogels include injectable hydrogels and non-injectable hydrogels. Injectable hydrogels are fluid and can be easily transported to the injured tissue. Importantly, formulations of injectable hydrogels have mechanical properties that greatly match the extracellular matrix (ECM) of spinal cord, which might promote axonal growth [27]. Several different kinds of injectable hydrogels are discussed below.

### Hydrogels acted on ECM

The cystic cavity formed after SCI is the main obstacle to CNS tissue repair while the cystic cavity is connected by a fibronectin-rich ECM [28]. Hydrogels can simulate the ECM in the cystic cavity to achieve the better therapeutic effect. The following two different hydrogels that act on the cellular matrix are mainly discussed.

Imidazole-polymer (I-5) hydrogel can promote ECM connectivity, which is injected to completely eliminate cysts in a rat model of SCI. Matrix metalloproteinase-9 expressed by macrophages in the fibrous ECM mediated fibrous ECM remodeling. A large number of macrophages exist in fibrous ECM. I-5 hydrogel promoted ECM remodeling by activating metalloproteinase-9. This dynamic interaction between I-5 hydrogel and macrophages resulted in the elimination of cystic cavities, neuronal repair, axonal increase and restoration in a rat model of SCI [28].

Decellularized tissue matrix (DTM) is a common biomaterial used for the repair of soft tissue. Moreover, as a bioactive component, ECM has been found in DTM and demonstrated that it can sustain high tissue specificity. Further continuous digestion, pH neutralization, and ion equalization of the DTM yield DTM hydrogels that can be applied in tissue engineering. DTM hydrogels have achieved good results in the SCI repair in recent years, mainly achieving the functions of filling the lesion cavity, promoting axonal growth and good immune regulation [29]. By comparing a decellularized tissue matrix hydrogel (DSCM-GEL) from the spinal cord with a decellularized tissue matrix hydrogel (DNM-GEL) from the peripheral nervous system, the results showed that DSCM-GEL kept the structure of ECM like nanofibers, enhanced the three-dimensional regenerative environment for the survival, proliferation and migration of neural stem cells and promoted the process of neuronal differentiation and synaptic formation of neural stem cells. It provided suitable conditions for endogenous cells to infiltrate into the injured site and it was conducive to promoting functional repair after SCI. Therefore, for future clinical transformation, the rational design of biological implants containing DTM hydrogel is much desirable for the successful recovery after SCI [29].

### Tauroursodeoxycholic acid (TUDCA) hydrogels

TUDCA is a hydrophilic acid that protects cells. A recent study has shown that TUDCA can be used as a replaceable drug based on its anti-neuro inflammatory effect in SCI rats [30]. However, the treatment effects were limited because of the use of intraperitoneal injections. Hydrogels, a high-water-content material, have been proposed as a drug delivery system that effectively offers a drug at a suitable targeted site in a short period. A research has found that direct injection of the TUDCA-hydrogel (TC gel) can decrease the amount of glial fibrillary acidic protein (GFAP) which is a marker of activated astrocyte to inhibit the inflammatory effect and promote functional recovery after SCI [30]. Moreover, hydrogels have no side effects, so the anti-inflammatory effect of TUDCA can be better developed.

### Peptide-modified hydrogels

Natural hydrogels have been widely developed for SCI repair in recent years. Hydrogels are usually modified by other materials to work better, such as the peptide. The integrin-binding peptide RGD, a tripeptide sequence containing arginine—glycine—aspartic acid, can be specifically recognized by integrin and can activate the pathway. It performs a key apoptotic function in cell cycle activity. The hydroxyphenyl derivative of HA (HA-PH), a promising HA-based material, can form covalently cross-linked hydrogel which has excellent mechanical properties. In a recent study, HA-PH was modified with peptide RGD to attain a soft injectable HA-PH-RGD hydrogel. The research has found that the hydrogel bridged the diseased cavity and enhanced axonal growth, which showed that the hydrogel might be a promising material in the repair of SCI [31]. In addition, a hydrogel modified with the platelet-derived growth factor (PDGF-A) and the RGD peptide promoted cell early survival and decreased teratoma formation [32].

### Self-assembling hydrogels

Peptide amphiphile hydrogels are absorbable, injectable, biofunctionalized and can control the release of nutrient factors [33]. Therefore, they have a good application prospect in regenerative medicine. Here, IKVAV functionalized PA hydrogel is taken as an example. A study found that injection of IKVAV functionalized PA hydrogel at the injured site inhibited glial scar formation and promoted axonal regeneration in SCI rats [33]. Then, scientists made further efforts to incorporate brain-derived neurotrophic factor (BDNF) into the self-assembled peptide hydrogels through different binding methods. They demonstrated that BDNF can be released continuously for 21 d while maintaining BDNF bioactivity through PA hydrogels. Also, injection of the BDNF-loaded IKVAV PA hydrogel induced inflammatory response after six weeks. Moreover, they also demonstrated that PA hydrogel can alleviate astrocyte proliferation and increase axonal preservation after severe SCI [33]. Therefore, the combined effect of this self-assembled peptide hydrogel and BDNF can reduce the barrier of a single treatment of neurotrophic factor and increase the sustained release [33, 34].

### Other hydrogels

Injectable hydrogels have potential application prospects in SCI regeneration. Apart from the ones described above, there are other types of injectable hydrogels. Silk fibroin/polydopamine hydrogel and heparin-Laponite hydrogel are mainly discussed.

The heparin-Laponite hydrogel is introduced at first. Fibroblast growth factor 4 (FGF4), a novel neuroprotective factor, is widely used for angiogenesis and stem cell differentiation [27]. A study showed that FGF4 may imitate the axonal regeneration of neurons by regulating the stabilization of microtubule after SCI, but injection of FGF4 is hardly effective in SCI because of the short half-life [27]. FGF4 belongs to FGF family containing a heparin-binding region, so it can be well combined with heparin. Laponite XLG, a 2-dimensional nanomaterial composed of nano-particles with good biodegradability and biocompatibility, is well used in regenerative medicine. This material can also be combined with other biomaterials such as hydrogel to form injectable nanocomposite hydrogels for SCI. A recent study has shown that a novel injectable Lap/Hep gel including FGF4 which was formed by heparin-FGF4 hybrid can regulate mitochondrial localization, enhance microtubule stability and axonal growth to promote recovery after SCI [27].

Then the injectable silk protein/polydopamine (SF/PDA) hydrogel will be introduced. Silk fibroin (SF), a water-insoluble protein with good biological and mechanical properties, has a wide application in nerve regeneration. Dopamine (DA), a kind of adhesion protein, can promote neuronal growth and differentiation. Therefore, DA has been widely concerned in biomedicine and mainly used for surface modification of different biomaterials. In recent years, the DA-based injectable hydrogels have shown a promising application in tissue regeneration. Based on the properties, in a recent study, an injectable SF/PDA composite hydrogel in different concentrations was formed. The addition of DA remarkably improved mechanical properties and physical properties and of the SF hydrogel. The study showed that SF/PDA hydrogel with the concentration of 2 mg/ml DA possessed the best repair effect on SCI, which indicated that SF/PDA hydrogels under this condition had potential application value in spinal cord regeneration [26].

## Tissue engineering

Hydrogel scaffolds can be greatly implanted into the SCI site for their good biocompatibility and biodegradability. Tissue engineering can improve SCI by promoting the formation of new neurons and axonal regeneration, inhibiting glial scar formation and decreasing secondary injury. Although hydrogel scaffolds have a good application prospect in tissue engineering, poor mechanical properties limited their applications. Therefore, recent research has focused on the preparation of composite scaffolds to improve the physical and chemical properties of scaffolds. The following mainly introduces composite scaffolds, several typical tissue engineering and combination therapy based on hydrogel scaffolds. Tissue engineering based on hydrogel scaffolds includes alginate hydrogel scaffolds, fibrous hydrogel scaffolds and combination therapy of hydrogel scaffolds combined with phototherapy, GFs, small molecules and stem cells.

### Composite scaffolds

Composite scaffolds are generally prepared by adding nanoparticles, fibers, nanotubes and clay minerals into hydrogels. They show significant promise in tissue engineering, especially when considering mechanical properties. Several materials for preparing composite hydrogels are introduced below.

#### Nanoparticles

The combination of hydrogels and nanoparticles has become a new research field in tissue engineering and regenerative medicine. There are many types of nanoparticles, including polymer nanoparticles (dendritic macromolecules and hyperbranched polyester), inorganic/ceramic nanoparticles (hydroxyapatite, silica, silicates and calcium phosphate) and metal/metal oxide nanoparticles (gold, silver and ferric oxide) [25]. Studies have shown that the addition of magnetic Fe_3_O_4_ nanoparticles into chitosan or polyethylene glycol hydrogel can promote the survival and differentiation of bone marrow mesenchymal stem cells [25, 35]. Hydroxyapatite was added to fibroin hydrogel to promote osteogenic differentiation of human bone marrow mesenchymal stem cells [35]. In addition, metal nanoparticles are one of the most attractive nanomaterials due to their corrosion resistance, oxidation resistance and other unique properties. They are characterized by high surface volume ratio, easy synthesis, surface chemistry and functionalization, which make them have a wide application in biomedicine. In recent years, Au and Ag nanoparticles have had potential applications in tissue regeneration. The combination of nanoparticles and hydrogels significantly improves the mechanical properties of hydrogels, thus overcoming the limitation of poor mechanical properties and enhancing the applicability of hydrogel scaffolds in tissue engineering.

#### Fibers

Studies have shown that adding fibers to bulk hydrogels can improve the mechanical properties of hydrogels [36, 37]. The structure of fibers is similar to the ECM. Hydrogels have high water content which is suitable for simulating soft tissue. The combination of fibers and hydrogels can produce a composite material similar to the natural microenvironment. The composite material has strong mechanical properties. Fiber–hydrogel composite is usually prepared by adding fibers to bulk hydrogels and layering the fibers with hydrogel. When fiber–hydrogel composite is applied to tissue engineering, the fiber density level in the hydrogels guides the cell behavior. Low-density fibers promote the production of ECM, while high-density fibers promote cell proliferation [36]. A recent study has reported a new type of nanofiber-hydrogel composite material used for the repair of SCI [37]. Its mechanical properties can be well-matched with the spinal cord nerve tissue. Different from ordinary fiber-hydrogel composite, this kind of composite material mainly relies on the unique bonding structure between fiber and hydrogel network interface, so as to maintain the mechanical integrity of the composite material at the damaged site, thus promoting the regeneration and repair of the damaged nerve tissue.

#### Nanotubes

Generally, carbon nanotubes have poor water dispersion and high costs. Halloysite nanotubes show a similar structure to carbon nanotubes and have many advantages, such as low toxicity, high biocompatibility, high dispersion and environmental protection. They have a wide application in the biomedical field. They can be dispersed into hydrogels due to their stable tubular morphology, unique charge distribution and crystal structure. The two materials can be combined to form a composite hydrogel by physical or chemical methods [38]. The composite material also has good applicability in tissue engineering. Studies have shown that the combination of nanotubes and chitosan has little effect on the pore structure, but increases the number of pores, which is conducive to the survival of cells [38, 39]. Naumenko and co-workers added halloysite nanotube into chitosan-gelatine-agarose hydrogels to form a nanocomposite hydrogel [39]. They observed that the mechanical strength of the composite hydrogel was improved by adding Hal nanotube. In addition, the biocompatibility and safe properties of the composite hydrogel were confirmed. Furthermore, the implantation of the nanocomposite hydrogel scaffold in rats promoted angiogenesis, which indicated the nanocomposite hydrogel scaffold was an ideal scaffold for tissue engineering.

#### Clay minerals

Clay minerals, known as sheet silicates, are inorganic layered nanomaterials with good biocompatibility and biodegradability. They are widely used in many fields. In medicine, they can promote wound healing and inhibit bleeding. They can also be applied as active ingredients in drug formulations. In pharmaceutical preparations, they can be applied as disintegrants, thinners, binders, thickeners and so on [40]. Clay minerals have been attracted much attention in recent years due to their layered structure and unique properties. The layered structures can be finely peeled to produce nanosheets with a high specific surface area. A number of studies have shown that adding clay minerals into the polymer can improve the mechanical properties and degradation of the polymer base [40, 41]. The composite hydrogels formed by adding clay minerals to hydrogels have strong mechanical properties. The effect of clay minerals on the properties of composite hydrogels depends on the type of clay minerals, the concentration of clay minerals and the dispersion of clay minerals in the polymer network structure of hydrogels. The physical and chemical interactions between polymers and clay minerals are the most important. Clay minerals can combine with macromolecular, such as polyacrylic acid and polyethylene glycol, polypeptide, alginate and chitosan to form nanocomposite hydrogels. A study has shown that many cells such as stem cells, fibroblasts and epithelial cells can achieve good adhesion, differentiation and proliferation on the nanocomposite hydrogels [41]. In addition, the mechanical properties of composite hydrogels containing clay minerals are also significantly enhanced, which indicates that the composite hydrogels have the potential in tissue engineering.

### Alginate hydrogel scaffolds

Alginate (ALG) is a biological substance that is constantly used to heal and regenerate human tissues extracted from brown seaweed. In addition, ALG has the ability to form hydrogels [42]. The network of ALG hydrogels is cross-linked by divalent cations such as Ca^2+^, Mg^2+^, Ba^2+^ and many others, which are vital to the function of ALG hydrogels [43]. The interaction of these cations fabricates ordered 3D structures of ALG hydrogels that are favorable for the hydrogel to form a scaffold loaded with drugs. And the physical properties of hydrogels are different from the different ions used. With high biocompatibility and external structural similarity to the ECM of living tissues, ALG hydrogels have been widely used in soft tissue engineering and have proven to be an effective biomaterial in SCI. Studies showed that implantation of soft ALG hydrogel at the injured site can improve functional recovery via the mechanisms of stabilization wound [43, 44], reduction secondary injury and inhibiting the formation of fibrous scarring.

Calcium-crosslinked ALG makes use of the electrostatic interaction of divalent cations with anionic polycarboxylate to form an ionic cross-linked network that can load small molecules, GFs or cells to maintain the regeneration of central nervous tissue [45]. Currently, Schwann cells (SCs) have been widely examined as an autologous cellular graft because they can support the axonal growth in SCI. Moreover, SCs also secrete neurotrophic factors to protect spared tissues, and one of the best representative neurotrophic factors is the BDNF. In a recent study, SC-seeded ALG hydrogels were grafted to the SCI site and BDNF expressing AAV5 was injected under the control of the tetracycline-regulated promoter. The study demonstrated that ALG hydrogels supported the survival of grafted SCs and promoted axonal growth. The number of axons that bridged the injured site significantly increased when BDNF expression was activated [46]. Based on the mentioned above, ALG hydrogels have a wide application prospect in SCI.

### Fibrous hydrogel scaffolds

In recent years, the hydrogel has been widely used as a bridge for the repair of SCI. They can not only promote the axonal growth and recovery of the damaged spinal cord, but also reduce and control inflammation. However, hydrogels are unstable in shape and easy to flow out of the SCI site, thus affecting the therapeutic effect. Based on this deficiency, researchers made the fibrous hydrogels to improve the strength of hydrogels by adding fiber, which had the ability to promote recovery and axonal regeneration [47]. Based on the fibrous hydrogel, there are nanofibers hydrogels and fibrin hydrogels, which are discussed below.

#### Nanofibers hydrogel scaffolds

Nanofibers, as a promising biomaterial, have the ability to imitate the architecture and the size scale of the natural ECM in spinal cord compared with the microfibers and microchannels which were previously used for SCI treatment. In previous work, researchers demonstrated that all kinds of drugs including low molecular weight lipophilic drugs could be incorporated into nanofiber matrices by electrospinning [37]. Collagen hydrogel enables sustained drug/gene delivery. A recent study showed that a biodegradable nanofibers-hydrogel scaffold which was composed of aligned poly electrospun nanofibers distributed in a three-dimensional structure within a collagen hydrogel could be as a biofunctionalized platform to provide sustained drug/gene delivery and contact guidance for SCI repair. The hydrogel scaffold promoted aligned axonal regeneration. In addition, no excessive scar tissue formed and inflammatory response was induced [47, 48]. Therefore, these results demonstrated the potential of nanofibers-hydrogel scaffold for the application of SCI repair.

#### Fibrin hydrogel scaffolds

Although hydrogels with excellent biocompatibility and biodegradability are widely applied in spinal cord regeneration, most of them are isotropic in structures. The white matter in spinal tissue composed of well-aligned bundles of axons, has hierarchical tissue structures from a nerve axon to a nerve fiber. While the aligned fiber structure was demonstrated that it was more similar to the nerve tissue, so it can be designed for nerve regeneration [49]. A pilot study of fibrin hydrogel showed that a new biomaterial-aligned fibrin hydrogel (AFG) can limit glial scarring, regenerate nerve fibers in the lesion site and have a beneficial influence in the improvement of injured axons to promote functional recovery [50]. In addition to hierarchically aligned structures, the stiffness of biomaterials is an essential feature of neural tissue regeneration. In a previous study, a hierarchically AFG with an aligned nanofiber structure and low resilience was developed to promote nerve fiber regeneration in SCI models [51, 52].

### Combination therapy

Effective treatments for SCI have not been developed due to its complex pathophysiological mechanism. This complexity suggests that multiple treatments may be more effective than the single treatment. Currently, hydrogel-based tissue engineering strategies involving scaffolds, phototherapy, cells, small molecules and GFs have provided wide prospects for the treatment of SCI. Hydrogels have a three-dimensional network structure and microenvironment that is similar to ECM, which can promote the cell growth and inserted biomacromolecules to maintain release. Table 1 is a summary of hydrogel scaffolds loaded with different GFs, small molecules and stem cells.

**Table 1:** Hydrogel scaffolds loaded with different GFs, small molecules and stem cells

GFs, small molecules and stem cells	Advantages	Disadvantages	Hydrogel-based delivery system
*GFs*			
BDNF [53–55]	Improving the axonal growth and promoting the formation of neural stem cells	Short half-life, low stability, and poor penetrativity of the BSCB	Hydrogel made by HA and MC
aFGF [56, 57]	Reducing the number of apoptotic neurons and the inflammatory reaction		A thermosensitive HP hydrogel loaded with aFGF
bFGF [56, 57]	Decreasing glial scar formation		A thermosensitive HP hydrogel loaded with bFGF
HGF [58]	Promoting neurogenesis and inhibiting glial scar formation		A gelatin-FA hydrogel scaffold loaded with HGF combined with a CBD
*Small molecules*			
Baricitinib [59]	Decreasing inflammation and promoting axonal regeneration	The first-pass effect; The special structure of BSCB and BBB	An injectable thermos-sensitive hydrogel loaded with baricitinib
Serpin [60, 61]	Decreasing inflammation and protecting neurons		A chitosan-collagen hydrogel loaded with serpin
Cab [62]	Reducing inhibitory scar formation and promoting axonal growth		A biodegradable and injectable hybrid hydrogel
MH, PTX [63, 64]	Anti-inflammatory and anti-apoptotic; Promoting axonal growth		A dual-drug delivery system based on alginate hydrogel
*Stem cells*			
*MSCs* [65–67]	Promoting neurogenesis, enhancing neuronal cell survival and inhibiting glial scar formation	Limited survival and low implantation rate and the failure of crossing the BBB	An agarose/carbomer-based hydrogel; A 3D gelatin sponge scaffold; Aligned fibrin hydrogel scaffold; Chitosan-based hydrogel
hMSCs [68, 69]	Promoting tissue regeneration and modulating the immune system		A three-dimensional hydrogel scaffold based on agarose/carbomer hydrogel
hUC-MSCs [70]	Abundant resources, convenient collection and low immunogenicity		A dual-enzymatically cross-linked gelatin hydrogel
Endometrium stem cells [71]	Low immunogenicity, high proliferation rate and the plasticity to differentiate into other cells		Fibrin hydrogel
IPSC-NPs [72, 73]	Altering the diseased environment		Optimized hydrogel based on gelatin
DPSCs [74–76]	Anti-inflammatory		HP hydrogel
OECs [77–79]	Creating a suitable environment		Collagen and fibrin hydrogels

*GFs* growth factors, *BDNF* brain-derived neurotrophic factors, *aFGF* acidic fibroblast growth factor, *bFGF* basic fibroblast growth factor, *HGF* hepatocyte growth factor, *BSCB* blood spinal cord barrier, *HA* hyaluronic acid, *MC* methylcellulose, *HP* heparin-poloxamer, *FA* furfueylamine, *CBD* collagen biding domain, *MH* minocvcline hydrochloride, *PTX* paclitaxel, *BBB* blood–brain barrier, *MSCs* mesenchymal stem cells, *hMSCs* human mesenchymal stem cells, *hUC-MSCs* human umbilical cord mesenchymal stem cells, *IPSC-NPs* induced pluripotent stem cell-derived neural progenitors, *DPSCs* dental pulp stem cells, *OECs* olfactory ensheathing cells

#### Hydrogel scaffolds loaded with GFs

SCI often leads to the failure of axonal regeneration. In recent years, GFs capable of instructing specific cellular responses in microenvironments have been demonstrated to promote axonal sprouting and regeneration, modulate the viability of damaged neurons and block inhibitory molecules, so they can be applied to treat SCI. However, GFs exist drawbacks, such as short half-life, low stability and poor penetrability of the blood spinal cord barrier (BSCB), which make it difficult to maintain long-term activity [56]. To solve the problem, GFs can be immobilized onto a hydrogel with excellent biocompatibility and biomechanical properties, which can be injected into the cystic cavity to regenerate a favorable microenvironment and promote the repair of damaged tissue. The most appropriate hydrogel for loading GFs should be a hydrogel that can act as a delivery system and maximize its potential in improving SCI. The following will mainly discuss several different GFs combined with hydrogels for SCI.

Nerve growth factor (NGF) can stimulate regeneration of injured axons, neurogenesis and angiogenesis. BDNF has been demonstrated that it is the most promising [56]. BDNF can improve the axonal growth and promote the proliferation of neural stem cells. BDNF can also be combined with hydrogel through chemical conjugation or physical absorption. Compared with physical absorption, chemical conjugation is more advantageous. Encapsulating BDNF into microspheres with good biodegradable and biocompatible can protect BDNF from microenvironmental damage and achieve sustained delivery at the injured sites. Poly (lactic-co-glycolic acid) (PLGA) microsphere has been widely applied in controlled release of BDNF. Studies have found that the implantation of composite hydrogel has been considered as a biofunctionalized delivery platform for neural regeneration and it can sustain the release of BDNF, such as HAMC-KAFAK/BDNF hydrogel, and HA-MC hydrogel. KAFAK was a biomaterial to control local inflammation after SCI [53–55]. HA-MC hydrogel was made by hyaluronic acid (HA), methylcellulose (MC) and a composite material containing BDNF-loaded poly PLGA particles for localized and sustained protein delivery.

Hepatocyte growth factor (HGF) which is a multipotent neurotrophic and neuroregenerative factor, can promote neurogenesis and inhibit glial scar formation. Moreover, immobilized HGF can be retained and localized at the injury site for longer periods than native HGF. In a recent study, HGF combined with a collagen-biding domain (CBD) was retained for 7 d in a gelatin-furfurylamine (FA) as a scaffold, which is longer than native HGF without the hydrogel scaffold [58].

Acidic fibroblast growth factor (aFGF) and basic fibroblast growth factor (bFGF) are powerful factors in the protection and regeneration of damaged neurons. aFGF can decrease the number of apoptotic neurons and the inflammatory reactions after SCI [56]. bFGF can reduce glial scar formation at later stage of SCI [57]. Studies have confirmed that the most suitable hydrogels for SCI was thermosensitive hydrogel, which has a high loading capacity and provides the GFs maximal protection [56, 57]. Researchers designed an innovative thermosensitive heparin-poloxamer (HP) hydrogel that can deliver GFs to the injured site to develop the aFGF-HP hydrogel and the GFs-HP hydrogel (GFs-HP) that consisted of bFGF and NGF. After injection of aFGF-HP and GFs-HP into the spinal cord lesion, they both showed sustained release of GFs and retained the activity of GFs [56, 57], which provided an effective treatment for SCI.

#### Hydrogel scaffolds loaded with small molecules

To date, many drugs have been used for SCI therapy. However, the delivery of therapeutic drugs to the CNS was a challenge because of the special structure of BSCB, blood–brain barrier (BBB) and the first-pass effect [59]. Moreover, drugs can be cleared rapidly due to CSF rapid renovation, which requires higher dosage/frequency. Therefore, it is important to prolong drug release at the SCI site. In the therapy of CNS diseases, hydrogels provide supporting substrates for tissue regeneration, and locally serve as a slow-release drug reservoir, which can reduce the frequency and total dose of administration, thereby reducing side effects and improving patient compliance. Several combination strategies of different representative drugs and hydrogels for SCI will be discussed in the following context.

Baricitinib, a JAK1/2 inhibitor with high efficiency, can decrease inflammation and promote axonal regeneration. A study has shown that PLGA/PEG block thermally reversible gelling polymers were widely used, especially for drug delivery due to their biodegradability and biocompatibility [59]. In addition, injectable hydrogels are thought to help with surgery, especially for those small, deep incisions. Based on this, researchers made an injectable PLGA-PEG-PLGA thermosensitive hydrogel scaffold loaded with baricitinib (Bari-P hydrogel). The results suggested that the hydrogel prolonged the baricitinib release, inhibited inflammation factors and reduced neuronal apoptosis [59].

Serine proteases can activate an acute inflammatory response to aggravate SCI. Serpin is an inhibitor of serine protease and an immunomodulatory biologic drug, which can decrease inflammation and protect neurons for SCI therapy. The combination of chitosan and a collagen hydrogel with stable structure produced a highly biodegradable material that had the properties of good biocompatibility and low antigenicity in animal models of SCI. Therefore, scientists demonstrated that serpin, when delivered by a chitosan-collagen hydrogel (CCH) into a rat of SCI, maintained the therapeutic effect and promoted functional recovery [60]. In addition, CCH loaded with selenium nanoparticle can also improve functional recovery [61].

Cabazitaxel (Cab), a microtubule inhibitor, is a second-line drug for the therapy of metastatic castrated prostate cancer. Researchers demonstrated that a low dose of Cab could decrease the formation of inhibitory scar and promote axonal growth by moderating microtubule stability [62]. However, the aqueous solubility of Cab is poor, so they designed a biodegradable and injectable hybrid hydrogel (Cab-M/H) scaffold to increase the aqueous solubility of Cab and established a SCI rat model. The results suggested that Cab-M/H hydrogel decreased fibrotic scarring and the axonal inhibitory factors, and boosted nerve regeneration to facilitate functional recovery [62].

It is necessary to combine multiple drugs to overcome several barriers owing to the multifaceted nature of SCI. Minocycline hydrochloride (MH), a neuroprotective agent, is a second-generation tetracycline used as an antibiotic in clinics with anti-inflammatory and anti-apoptotic properties. Paclitaxel (PTX), an anti-cancer drug, can also promote axonal growth at the trauma site by stabilizing microtubules. Scientists assumed that the combination of MH and PTX would be a promising therapy for SCI repair. Considering the interaction of MH with metal ions, they made affinity alginate hydrogel scaffold as a delivery system to avoid the risk of hepatotoxity and even death caused by MH and toxicity of PTX on peripheral nerve tissue. The hydrogel based on metal-ion chelation and electrostatic interaction incorporated MH and PTX which were incorporated into PLGA microspheres and inserted into the alginate hydrogel to form a dual-drug delivery system. The system decreased inflammation and promoted neuronal regeneration [63]. Furthermore, the therapy of agents combined GFs for SCI is also a promising method [64].

#### Hydrogel scaffolds loaded with stem cells

In recent years, cell-based therapy strategy on SCI has achieved a good effect. The implant of stem cells and hydrogel scaffolds is a promising approach for SCI repair. However, there are many challenges in the cell therapy approach such as migration to the target site, attachment to the SC surface and cell survival in CSF [65]. One strategy to over these drawbacks is the implantation of stem cells and the hydrogel scaffolds which can promote tissue regeneration by releasing neuro-protective factors. Strategies based on different stem cells and hydrogel scaffolds will be discussed below.

Regenerative medicine based on mesenchymal stem cells (MSCs) is regarded as a promising strategy to repair SCI tissue. MSCs, as a particularly promising therapeutic strategy, can promote neurogenesis, enhance neuronal cell survival and inhibit glial scar formation. However, recent studies have found that the survival of cells after implantation was limited and the implantation rate was low, because implanted cells did not survive for a long time and the survival rate was greatly reduced when the cells were implanted in a damaged and highly reactive environment. Hydrogels, a promising biomaterial for SCI, can be as cell carriers to enhance both cell survival and implantation rate in the injured site. These hydrogels have proved better effect [65–67]: (1) An agarose/carbomer-based hydrogel can offer a suitable microenvironment to sustain the survival of MSCs; (2) A 3D gelatin sponge scaffold loaded with MSCs can improve axonal regeneration; (3) The AFG scaffold can control neural differentiation; (4) Chitosan-based hydrogel with anti-inflammatory and anti-oxidant properties can decrease inflammatory factors to create a suitable environment for MSCs.

Human mesenchymal stem cells (hMSCs) as an effective therapeutic approach in tissue repair after SCI, can promote tissue regeneration by releasing exogenous factors and modulating the immune system. However, direct injection may result in limited survival of stem cells and limited therapeutic effect since cells cannot cross the BBB and the ischemia environment in the injured area after SCI [68, 69]. A method can be used to overcome the problem by encapsulating hMSCs into a new three-dimensional hydrogel scaffold based on agarose/carbomer hydrogel.

Human umbilical cord mesenchymal stem cells (hUC-MSCs) are less immunogenic, which have the properties of abundant resources, convenient collection and low immunogenicity [70]. However, the effect of stem cell therapy is still hindered by low cell implantation rate, low survival rate, and uncontrolled cell differentiation. Based on the mentioned above, scientists designed the dual-enzymatically cross-linked gelatin hydrogel as an injectable scaffold composed of hydrogen horseradish peroxidase (HRP) and galactose oxidase (GalOx) loaded with hUC-MSCs. The results showed that implantation of the hydrogel system was a promising therapy strategy for SCI [70].

Endometrium stem cells are superior to other MSCs in some characteristics including low immunogenicity, high proliferation rate and the plasticity to differentiate into other cells [74]. Considering the complexity and sensitivity of CNS, hydrogel scaffolds with excellent biocompatible and biodegradable can be regarded as an ideal biomaterial. A recent study showed transplantation of human endometrial stem cells encapsulated in fibrin hydrogel reduced glial scar formation and enhanced axonal regeneration in a SCI rat model, which suggested that the strategy could be used for SCI recovery [71].

With the application of the induced pluripotent stem cell (IPSC) method, IPSCs have been a better cell type for the treatment of SCI [72]. Progenitor cells and stem cells are particularly helpful tools in regenerative medicine because they can rebuild the diseased environment. In general, they can increase the level of neurotrophic factors to promote nerve regeneration. IPSC-derived neural progenitors (IPSC-NPs) for the treatment of acute SCI are positive through forming glial scar, promoting axonal growth and sparing tissue to facilitate spinal cord regeneration. IPSC-NPs can be combined with hydrogels that are composed of the gelatin combined with methacrylate (GelMA) and laminin-coated pHEMA-MOETACL (LHM) hydrogel [72, 73], which can offer a suitable environment for the cell growth to treat SCI rat model.

Stem cells transplantation can provide new neural cells in the replacement of dead cells and generate many trophic factors. Dental stem cells derived from stem cells possess stem cell characteristics. Therefore, they can also be applied for SCI to promote tissue regeneration. Besides, they have the ability to decrease inflammatory factors and promote motor functional recovery through releasing trophic factors [74]. In a study, an innovative thermosensitive HP hydrogel including bFGF and dental pulp stem cells (DPSCs) was designed to be transported to the site of SCI to retain the high density of DPSCs and the continued role of bFGF in the process of recovery. The results showed that the implant of HP hydrogel including DPSCs and bFGF promoted nerve regeneration and functional recovery [74, 75]. The human gingival mesenchymal stem cells are derived from DPSCs [76], so it can also be applied in SCI treatment.

Olfactory ensheathing cells (OECs) are an important and clinically relevant cell transplantation population for SCI, and they can produce a suitable environment for axonal growth in the damaged CNS [77]. Studies have indicated that both collagen and fibrin hydrogels could improve the delivery [78, 79], survival and retention of transplanted OECs for SCI.

#### Hydrogel scaffolds combined with phototherapy

Hydrogels can mimic the ECM microenvironment very well. Phototherapy as a promising strategy can further promote hydrogels to imitate the dynamic and complex nature of the ECM because it has the property to provide dosage controls [80]. The combination of hydrogels and phototherapy is more effective for treating SCI. The following are two examples of hydrogels that combine phototherapy with hydrogels. They are photosensitive hydrogels and photo-crosslinked hydrogels.

SCI often leads to the loss of cell viability and lack of directional control of neuronal regeneration. A study has confirmed that the introduction of nerve guidance conduits (NGCs) not only regulated the internal microenvironment to promote cell regeneration but also guided the regeneration of nerve endings, which was a good treatment for SCI [80]. However, the disadvantage was that most synthetic NGC scaffolds lack the ability of cell adhesion due to the absence of natural recognition sites, resulting in low viability. A study has constructed a microporous functional hydrogel (MFH) scaffold with a guide conduit and a photo-responsive monomer was copolymerized for protein coupling [80]. Proteins were effectively located on the whole inner surface of the hydrogel while also reducing the possibility of light attenuation. After the hydrogel scaffold was implanted into the transected spinal cord, it was found that the optimized hydrogel scaffold could improve the motor recovery of rats after SCI. Histology further demonstrated that the design of the hydrogel scaffold provided a favorable biologic site for neurons to generate directional neuronal tissue and facilitated the repair of SCI [80]. The proposed design of photosensitive hydrogel provides another good option for the treatment of SCI.

Inflammation is commonly associated with SCI. Microglia/macrophages are the main contributors to inflammation factors. Moreover, activated microglia/macrophages can produce pro-inflammatory mediators to inhibit the repair of spinal cord and homeostasis. Spinal cord lacks of lymphatic circulation against invasion of inflammatory mediators which can aggravate SCI. Therefore, SCI can be treated by eliminating inflammation by removing inflammatory cells and reducing pro-inflammatory factors. The photo-crosslinked gelatin 3D hydrogel system applied at present with good biocompatibility and biodegradation can simulate the microenvironment of ECM in the spinal cord and suppress inflammatory factors. However, it can only work in the injured site because of the lack of target specificity. In addition, the system requires photoactivation. PLX3397, an inhibitor of colony stimulating factor 1 receptor (CSF1R), can eliminate microglia and rebuilt microglial population. The treatment can alleviate persistent inflammation caused by brain injury. A study showed that the treatment of the implantation of photo-crosslinked gelatin 3D hydrogel and PLX3397 reduced the number of activated microglia/macrophages and facilitated the formation of new neurons to promote SCI repair [81, 82].

## Discussion

SCI is a severe traumatic disease of the CNS, which will bring a great influence on the patients’ lifestyle and cause certain pressure on the patients’ physical and psychological aspects. The introduction of hydrogels as a biomaterial has opened up a new way for the treatment of induced regeneration after SCI. Hydrogels are a kind of highly hydrated material with water molecules and hydrophilic polymer networks, which have a particularity similar to nerve tissue. They have the ability to fill the cystic cavity and promote axonal growth and cell differentiation [83]. Hydrogels can be implanted or injected at the site of the lesion and corresponding therapies were injectable hydrogel therapy and tissue engineering introduced in this article.

The injectable hydrogels introduced in this paper include hydrogels acted on ECM, TUDCA hydrogels, peptide-modified hydrogels and self-assembling hydrogels. Tissue engineering includes alginate hydrogel scaffolds, fibrous hydrogel scaffolds and combination therapy based on hydrogel scaffolds combined with phototherapy, GFs and stem cells. Hydrogels used to treat SCI should mimic the mechanical properties of nerve tissue to promote the regeneration of damaged tissue [83]. Injectable hydrogels are fluid and can be easily transported to the injured tissue. Importantly, formulations of injectable hydrogels have the mechanical properties that greatly match the ECM of spinal cord, which might promote axonal growth [34]. When using injectable hydrogels for the treatment of SCI, it is necessary to consider the viscosity, formulation, type of hydrogels and other materials bound to hydrogels. Different types of hydrogels have different properties, such as mechanical properties, viscosity, biocompatibility, biodegradation and so on. In order to greatly match the ECM of the spinal cord and achieve better therapeutic effect, the performance of hydrogels can be improved by modifying hydrogels or preparing composite hydrogels. Hydrogels acting on ECM can treat SCI by promoting ECM remodeling and eliminating cystic cavity [28, 29]. TUDCA hydrogels are used to treat SCI through anti-inflammatory activity [30]. Compared with the two injectable hydrogels mentioned above, peptide-modified hydrogels and self-assembling hydrogels have certain advantages. These hydrogels have better biocompatibility and can promote cell survival and SCI recovery in many ways [31–34]. But injectable hydrogels also have their drawbacks, they are unstable in shape and easy to flow out of the SCI site due to their characteristics, thus influencing the therapeutic effect. Compared with injectable hydrogels, tissue engineering has a wider application prospect. Hydrogel scaffolds as an excellent and multi-function platform can be further combined with other therapeutic substances, such as drugs, GFs and stem cells. They can protect molecules or cells from enzyme degradation or adverse immune response and increase the potential role of transplanted cells [83]. However, the mechanical properties of hydrogel scaffolds are important when they are considered for implantation. Hydrogel scaffolds for SCI have an inherent limitation of strength and mechanical properties. Thus they are usually supplemented with “miscellaneous” materials forming composites to improve their properties. For example, adding fiber or SF to the hydrogel scaffolds can increase the hardness of the hydrogel scaffolds, adding natural hydrogels to the synthetic hydrogels can improve the degradability of the synthetic hydrogels, adding a light initiator to the hydrogel scaffolds can achieve photopolymerization to solidify their shape.

ALG hydrogel scaffolds with good biocompatibility are similar to the structure of ECM of living tissue, which is a kind of effective biological material for the treatment of SCI. Studies have shown that the implantation of a soft ALG hydrogel into the injury site can reduce secondary injury by stabilizing the wound surface and inhibiting fibrous scar formation to promote functional recovery [43, 44]. The fibrous hydrogels have strong hardness and have the ability to promote the recovery and axonal regeneration [47]. Compared with two kinds of hydrogel scaffolds mentioned above, the combination therapy based on hydrogel scaffolds has a better therapeutic effect. When hydrogel scaffolds are combined with phototherapy, phototherapy as a promising strategy can further promote hydrogels to imitate the dynamic and complex nature of the ECM because it has the property to provide dosage controls [80]. GFs capable of instructing specific cellular responses in microenvironments have been demonstrated to promote axonal sprouting and regeneration, modulate the viability of damaged neurons and block inhibitory molecules [56, 82]. MSCs, as a particularly promising therapeutic tool, can promote neurogenesis, enhance neuronal cell survival and inhibit glial scar formation [65]. At present, compared with other therapies, tissue engineering based on stem cells and GFs has a promising application prospect. But it is necessary to design a suitable hydrogel scaffold to deliver stem cells and GFs to the targeted site. The suitable hydrogel scaffold can also increase the survival rate of transplanted cells and prolong the release time to achieve a good treatment effect. The mechanical properties, viscosity, cellular compatibility, degradability and other properties of hydrogels should be taken into consideration when selecting suitable hydrogel scaffolds. Tissue engineering is usually combined with different materials to form composite hydrogel scaffolds, thus improving its performance. Studies have confirmed that the most suitable hydrogel scaffold for SCI was thermosensitive hydrogel, which has high loading capacity, provides the GFs maximal protection and a suitable living environment for stem cells and plays a good therapeutic role [56, 57]. Although many studies of cell-based tissue engineering using small animal models of SCI have yielded promising results, there have been no human trials of hydrogels for SCI so far. In order to successfully transform the experiment into a clinical trial, successful amplification in large animal models must first be demonstrated and this is what future researchers need to do further.

## Conclusions

This paper mainly summarizes multimodal therapy strategies based on hydrogels for the repair of SCI. The therapy model of different types of hydrogels which are served as biological scaffolds and combined with other nutritional factors, small molecules and various stem cells for SCI is more common now.

An effective therapeutic strategy for SCI must combine multiple factors to solve every challenge in a coordinated manner due to the complex pathophysiology of SCI, such as stem cells, anti-inflammatory drugs, and GFs, all of which require hydrogels served as scaffolds to deliver into the injured site. However, hydrogels are unstable in shape and easy to flow out of the SCI site due to their characteristics, thus influencing the therapeutic effect. Therefore, it is necessary to further optimize the structure of hydrogels, such as adding fiber or preparing composite hydrogels to improve the strength of hydrogels. Moreover, most of the current studies have been conducted in SCI models, but it is unknown whether the same effect will be seen in the clinic.

Hydrogels with excellent biocompatibility and biodegradability are considered as a bridge for post-SCI repair. They can well imitate the ECM of the spinal cord microenvironment. Therefore, hydrogels have a better application prospect in treating SCI in the future.

## Data Availability

Not applicable.

## Abbreviations

AFG: Aligned fibrin hydrogel
aFGF: Acidic fibroblast growth factor
ALG: Alginate
BBB: Blood–brain barrier
BDNF: Brain-derived neurotrophic factor
bFGF: Basic fibroblast growth factor
BSCB: Blood spinal cord barrier
Cab: Cabazitaxel
CBD: Collagen-biding domain
CCH: Chitosan-collagen hydrogel
CNS: Central nervous system
CSF: Cerebrospinal fluid
CSF1R: Colony stimulating factor 1 receptor
DA: Dopamine
DNM-GEL: Decellularized tissue matrix hydrogel from the peripheral nervous system
DPSCs: Dental pulp stem cells
DSCM-GEL: Decellularized tissue matrix hydrogel from the spinal cord
DTM: Decellularized tissue matrix
ECM: Extracellular matrix
FA: Furfurylamine
FGF4: Fibroblast growth factor 4
GalOx: Ggalactose oxidase
GFAP: Glial fibrillary acidic protein
GFs: Growth factors
HA: Hyaluronic acid
HGF: Hepatocyte growth factor
hMSCs: Human mesenchymal stem cells
HP: Heparin-poloxamer
HRP: Horseradish peroxidase
hUC-MSCs: Human umbilical cord mesenchymal stem cells
I-5 hydrogel: Imidazole-polymer hydrogel
IPSC-NPs: Induced pluripotent stem cell-derived neural progenitors
MC: Methylcellulose
MFH: Microprous functional hydrogel
MH: Minocycline hydrochloride
MSCs: Mesenchymal stem cells
NGCs: Nerve guidance conduits
NGF: Nerve growth factor
OECs: Olfactory ensheathing cells
PDGF: Platelet-derived growth factor
PTX: Paclitaxel
SCI: Spinal cord injury
SCs: Schwann cells
SF: Silk fibroin
TUDCA: Tauroursodeoxycholic acid
